# Supplementary material for: Identification of photoperiod-induced specific miRNAs in the adrenal glands of Sunite sheep (Ovis aries)
Source: Front Vet Sci. 2022 Jul 22;9:888207. doi: 10.3389/fvets.2022.888207 (PMC9354845; doi:10.3389/fvets.2022.888207)
Supplement: Supplementary file 1 [file Data_Sheet_1.ZIP › Supplementary file/miRDeep2 file.zip!/miRDeep2 file/Know_Structure/LP42A2/oar-mir-487b.pdf]

miRBase precursor : oar-mir-487b
Total read count : 2675
oar-miR-487b-5p read count : 50
oar-miR-487b-3p read count : 2618
remaining reads : 7

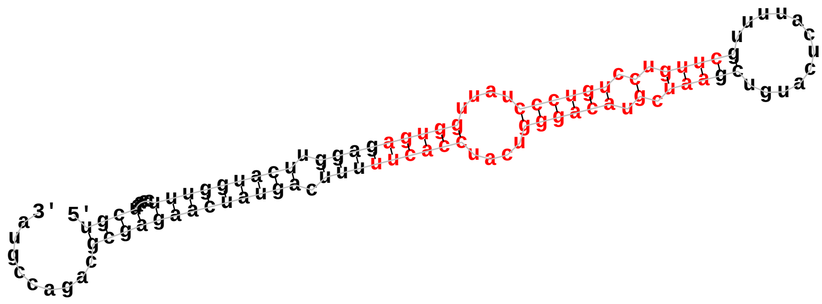

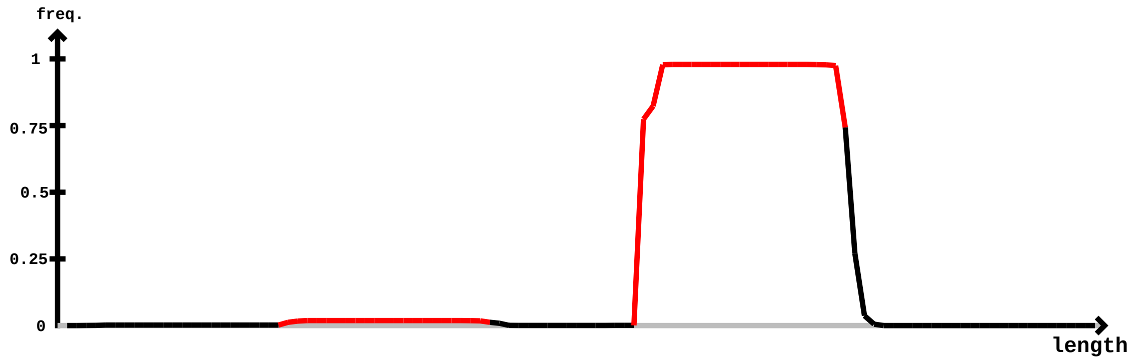


```
                                                            oar-miR-487b-3p
                      oar-miR-487b-5p
5'- ugcuaaccuuugguacuuggagagugguuaucccuguccuguucguuuuacucaugucgaaucguacagggucauccacuuuuucaguaucaagagcgcagaccgua -3'  exp
    .(((....((((((((.((((((((....((((((.(.(((((............))))).).))))))....)))))))).)))))))))))..............      reads  mm  sample
    .gcuaaccuuugguacuuggaga....................................................................................      1      0   seq
    ..cuaaccuuugguacuuggaga....................................................................................      1      0   seq
    ...uaaccuuugguacuuggag.....................................................................................      1      0   seq
    ...uaaccuuugguacuuggaga....................................................................................      2      0   seq
    ......................agugguuaucccuguccug..................................................................      1      0   seq
    ......................agugguuaucccuguccugu.................................................................      2      0   seq
    ......................agugguuaucccuguccuguu................................................................      15     0   seq
    ......................agugguuaucccuguccuguuc...............................................................      7      0   seq
    ......................agugguuaucccuguccuguucg..............................................................      4      0   seq
    .......................gugguuaucccuguccuguuc...............................................................      2      0   seq
    .......................gugguuaucccuguccuguucg..............................................................      13     0   seq
    .......................gugguuaucccuguccuguucgu.............................................................      1      0   seq
    ........................ugguuaucccuguccuguuc...............................................................      1      0   seq
    ........................ugguuaucccuguccuguucg..............................................................      4      0   seq
    ...........................................cguuuuacucaugucgaa..............................................      1      0   seq
    ........................................................ucgaaucguacagggucaucc..............................      1      0   seq
    ...........................................................aaucguacagggucaucca.............................      2      0   seq
    ...........................................................aaucguacagggucauccac............................      9      0   seq
    ...........................................................aaucguacagggucauccacu...........................      598    0   seq
    ...........................................................aaucguacagggucauccacuu..........................      1202   0   seq
    ...........................................................aaucguacagggucauccacuuu.........................      256    0   seq
    ...........................................................aaucguacagggucauccacuuuu........................      1      0   seq
    ............................................................aucguacagggucauccacu...........................      15     0   seq
    ............................................................aucguacagggucauccacuu..........................      37     0   seq
    ............................................................aucguacagggucauccacuuu.........................      71     0   seq
    ............................................................aucguacagggucauccacuuuu........................      9      0   seq
    .............................................................ucguacagggucauccacu...........................      7      0   seq
    .............................................................ucguacagggucauccacuu..........................      24     0   seq
    .............................................................ucguacagggucauccacuuu.........................      296    0   seq
    .............................................................ucguacagggucauccacuuuu........................      75     0   seq
    .............................................................ucguacagggucauccacuuuuu.......................      14     0   seq
    ..............................................................cguacagggucauccacuuu.........................      2      0   seq
```
